# Brain alterations in the early Alzheimer’s continuum with amyloid-β, tau, glial and neurodegeneration CSF markers

**DOI:** 10.1093/braincomms/fcac134

**Published:** 2022-05-24

**Authors:** Gemma Salvadó, Mahnaz Shekari, Carles Falcon, Grégory Operto, Marta Milà-Alomà, Gonzalo Sánchez-Benavides, Raffaele Cacciaglia, Eider Arenaza-Urquijo, Aida Niñerola-Baizán, Andrés Perissinotti, Carolina Minguillon, Karine Fauria, Gwendlyn Kollmorgen, Ivonne Suridjan, José Luis Molinuevo, Henrik Zetterberg, Kaj Blennow, Marc Suárez-Calvet, Juan Domingo Gispert, ALFA Study, Annabella Beteta, Anna Brugulat-Serrat, Alba Cañas, Irene Cumplido, Carme Deulofeu, Ruth Dominguez, Maria Emilio, Sherezade Fuentes, José María González-de-Echavarri, Oriol Grau-Rivera, Laura Hernandez, Gema Huesa, Jordi Huguet, Iva Knezevic, Paula Marne, Tania Menchón, Maria Pascual, Albina Polo, Sandra Pradas, Aleix Sala-Vila, Anna Soteras, Laia Tenas, Marc Vilanova, Natalia Vilor-Tejedor

**Affiliations:** Barcelonaβeta Brain Research Center (BBRC), Pasqual Maragall Foundation, Barcelona, Spain; IMIM (Hospital del MarMedical Research Institute), Barcelona, Spain; Barcelonaβeta Brain Research Center (BBRC), Pasqual Maragall Foundation, Barcelona, Spain; IMIM (Hospital del MarMedical Research Institute), Barcelona, Spain; Universitat Pompeu Fabra, Barcelona, Spain; Barcelonaβeta Brain Research Center (BBRC), Pasqual Maragall Foundation, Barcelona, Spain; IMIM (Hospital del MarMedical Research Institute), Barcelona, Spain; Centro de Investigación Biomédica en Red Bioingeniería, Biomateriales y Nanomedicina, (CIBER-BBN), Barcelona, Spain; Barcelonaβeta Brain Research Center (BBRC), Pasqual Maragall Foundation, Barcelona, Spain; IMIM (Hospital del MarMedical Research Institute), Barcelona, Spain; Centro de Investigación Biomédica en Red de Fragilidad y Envejecimiento Saludable (CIBERFES), Instituto de Salud Carlos III, Madrid, Spain; Barcelonaβeta Brain Research Center (BBRC), Pasqual Maragall Foundation, Barcelona, Spain; IMIM (Hospital del MarMedical Research Institute), Barcelona, Spain; Universitat Pompeu Fabra, Barcelona, Spain; Centro de Investigación Biomédica en Red de Fragilidad y Envejecimiento Saludable (CIBERFES), Instituto de Salud Carlos III, Madrid, Spain; Barcelonaβeta Brain Research Center (BBRC), Pasqual Maragall Foundation, Barcelona, Spain; IMIM (Hospital del MarMedical Research Institute), Barcelona, Spain; Centro de Investigación Biomédica en Red de Fragilidad y Envejecimiento Saludable (CIBERFES), Instituto de Salud Carlos III, Madrid, Spain; Barcelonaβeta Brain Research Center (BBRC), Pasqual Maragall Foundation, Barcelona, Spain; IMIM (Hospital del MarMedical Research Institute), Barcelona, Spain; Centro de Investigación Biomédica en Red de Fragilidad y Envejecimiento Saludable (CIBERFES), Instituto de Salud Carlos III, Madrid, Spain; Barcelonaβeta Brain Research Center (BBRC), Pasqual Maragall Foundation, Barcelona, Spain; IMIM (Hospital del MarMedical Research Institute), Barcelona, Spain; Centro de Investigación Biomédica en Red de Fragilidad y Envejecimiento Saludable (CIBERFES), Instituto de Salud Carlos III, Madrid, Spain; Centro de Investigación Biomédica en Red Bioingeniería, Biomateriales y Nanomedicina, (CIBER-BBN), Barcelona, Spain; Nuclear Medicine Department, Hospital Clínic Barcelona, Barcelona, Spain; Centro de Investigación Biomédica en Red Bioingeniería, Biomateriales y Nanomedicina, (CIBER-BBN), Barcelona, Spain; Nuclear Medicine Department, Hospital Clínic Barcelona, Barcelona, Spain; Barcelonaβeta Brain Research Center (BBRC), Pasqual Maragall Foundation, Barcelona, Spain; IMIM (Hospital del MarMedical Research Institute), Barcelona, Spain; Centro de Investigación Biomédica en Red de Fragilidad y Envejecimiento Saludable (CIBERFES), Instituto de Salud Carlos III, Madrid, Spain; Barcelonaβeta Brain Research Center (BBRC), Pasqual Maragall Foundation, Barcelona, Spain; Centro de Investigación Biomédica en Red de Fragilidad y Envejecimiento Saludable (CIBERFES), Instituto de Salud Carlos III, Madrid, Spain; Roche Diagnostics GmbH, Penzberg, Germany; Roche Diagnostics International Ltd, Rotkreuz, Switzerland; Barcelonaβeta Brain Research Center (BBRC), Pasqual Maragall Foundation, Barcelona, Spain; Department of Psychiatry and Neurochemistry, Institute of Neuroscience and Physiology, University of Gothenburg, Mölndal, Sweden; Clinical Neurochemistry Laboratory, Sahlgrenska University Hospital, Mölndal, Sweden; Department of Neurodegenerative Disease, UCL Institute of Neurology, Queen Square, London, United Kingdom; UK Dementia Research Institute at UCL, London, United Kingdom; Department of Psychiatry and Neurochemistry, Institute of Neuroscience and Physiology, University of Gothenburg, Mölndal, Sweden; Clinical Neurochemistry Laboratory, Sahlgrenska University Hospital, Mölndal, Sweden; Barcelonaβeta Brain Research Center (BBRC), Pasqual Maragall Foundation, Barcelona, Spain; IMIM (Hospital del MarMedical Research Institute), Barcelona, Spain; Centro de Investigación Biomédica en Red de Fragilidad y Envejecimiento Saludable (CIBERFES), Instituto de Salud Carlos III, Madrid, Spain; Servei de Neurologia, Hospital del Mar, Barcelona, Spain; Barcelonaβeta Brain Research Center (BBRC), Pasqual Maragall Foundation, Barcelona, Spain; IMIM (Hospital del MarMedical Research Institute), Barcelona, Spain; Centro de Investigación Biomédica en Red Bioingeniería, Biomateriales y Nanomedicina, (CIBER-BBN), Barcelona, Spain

**Keywords:** structural magnetic resonance imaging, fluorodeoxyglucose positron emission tomography, cerebrospinal fluid biomarkers, neurodegeneration, neuroinflammation

## Abstract

Higher grey matter volumes/cortical thickness and fluorodeoxyglucose uptake have been consistently found in cognitively unimpaired individuals with abnormal Alzheimer’s disease biomarkers compared with those with normal biomarkers. It has been hypothesized that such transient increases may be associated with neuroinflammatory mechanisms triggered in response to early Alzheimer’s pathology. Here, we evaluated, in the earliest stages of the Alzheimer’s *continuum*, associations between grey matter volume and fluorodeoxyglucose uptake with CSF biomarkers of several pathophysiological mechanisms known to be altered in preclinical Alzheimer’s disease stages. We included 319 cognitively unimpaired participants from the ALFA+ cohort with available structural MRI, fluorodeoxyglucose PET and CSF biomarkers of amyloid-β and tau pathology (phosphorylated tau and total tau), synaptic dysfunction (neurogranin), neuronal and axonal injury (neurofilament light), glial activation (soluble triggering receptor on myeloid cells 2, YKL40, GFAP, interleukin-6 and S100b) and α-synuclein using the Roche NeuroToolKit. We first used the amyloid-β/tau framework to investigate differences in the neuroimaging biomarkers between preclinical Alzheimer’s disease stages. Then, we looked for associations between the neuroimaging markers and all the CSF markers. Given the non-negative nature of the concentrations of CSF biomarkers and their high collinearity, we clustered them using non-negative matrix factorization approach (components) and sought associations with the imaging markers. By groups, higher grey matter volumes were found in the amyloid-β-positive tau-negative participants with respect to the reference amyloid-β-negative tau-negative group. Both amyloid-β and tau-positive participants showed higher fluorodeoxyglucose uptake than tau-negative individuals. Using the obtained components, we observed that tau pathology accompanied by YKL-40 (astrocytic marker) was associated with higher grey matter volumes and fluorodeoxyglucose uptake in extensive brain areas. Higher grey matter volumes in key Alzheimer-related regions were also found in association with two other components characterized by a higher expression of amyloid-β in combination with different glial markers: one with higher GFAP and S100b levels (astrocytic markers) and the other one with interleukin-6 (pro-inflammatory). Notably, these components’ expression had different behaviours across amyloid-β/tau stages. Taken together, our results show that CSF amyloid-β and phosphorylated tau, in combination with different aspects of glial response, have distinctive associations with higher grey matter volumes and increased glucose metabolism in key Alzheimer-related regions. These mechanisms combine to produce transient higher grey matter volumes and fluorodeoxyglucose uptake at the earliest stages of the Alzheimer’s *continuum*, which may revert later on the course of the disease when neurodegeneration drives structural and metabolic cerebral changes.

## Teaser

Salvadó *et al.* show that amyloid-β and tau pathologies, in combination with different aspects of glial response, have distinctive associations with brain’s structure and function in key Alzheimer-related regions. These mechanisms combine to produce transient higher grey matter volumes and glucose metabolism at the earliest stages of the Alzheimer’s continuum.

## Graphical Abstract

**Graphical abstract:**
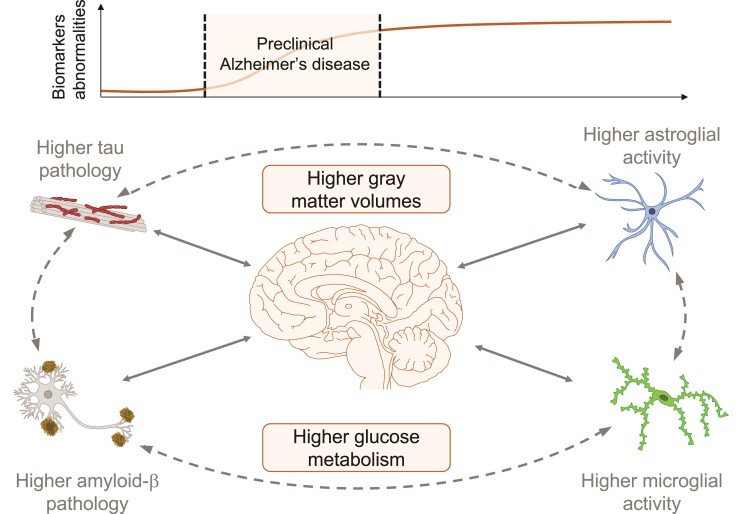

## Introduction

From a research perspective, Alzheimer’s disease is defined as the presence of abnormal biomarkers of amyloid-β (Aβ) and tau, which constitute the biological hallmarks of the disease.^1^ Individuals with altered Aβ biomarkers but normal tau biomarkers are defined as to show ‘Alzheimer’s pathological change’, which is considered the earliest phase of the ‘Alzheimer’s *continuum*’. Well-established biomarkers for Aβ and tau include CSF and PET measurements. Measures of neurodegeneration or neuronal injury are MRI, fluorodeoxyglucose ([^18^F]FDG) PET, as well as CSF neurofilament light (NfL). However, neurodegeneration markers are not considered to be specific for Alzheimer’s disease as they may be altered due to a variety of underlying aetiologies. Therefore, under this research framework, markers of neurodegeneration, as well as cognition, are used only to stage severity but not to define Alzheimer’s disease.

Even though alterations in Aβ and tau define Alzheimer’s disease, other pathophysiological pathways are also affected early in the Alzheimer’s *continuum*. A better understanding of these alterations, their interrelationships and when they emerge in the Alzheimer’s *continuum* would provide additional information on the pathophysiology of the disease and inform the rational development of disease modifying interventions. It is of particular interest to inform preventive interventions, to identify biological pathways that may eventually lead to neurodegeneration and that are already altered in asymptomatic Alzheimer’s disease stages.

In a previous study in cognitively unimpaired individuals, we found that just after CSF Aβ42/40 becomes abnormal, there is a steep increase in CSF biomarkers of tau-related [phosphorylated tau (p-tau) and total tau (t-tau)] and synaptic dysfunction (neurogranin) and, to a lesser extent, in neuronal and axonal injury (NfL) and glial markers [soluble triggering receptor on myeloid cells 2 (sTREM2), YKL40, and glial fibrillary acidic protein (GFAP)].^2^ In a follow-up work, we reported that these markers were also associated with cerebral Aβ deposition as measured by PET.^3^ However, the relationship between these CSF biomarkers and structural and metabolic alterations in the brain are not well understood, especially in these early phases of the disease.

Neurodegeneration is present in the late stages of the Alzheimer’s *continuum*; however, brain alterations in the opposite direction have been described in earlier phases of the disease. In particular, increased grey matter (GM) volume and/or cortical thickness has been found with the earliest abnormalities of Aβ in cross-sectional,^4–8^ as well as in longitudinal studies.^9^ In addition, increments in glucose metabolism, as measured with [^18^F]FDG PET, have also been shown in association with Aβ burden in cognitively unimpaired participants.^4,10^ Although consistently reported, the biological reasons for these unexpected increments are still not known. Some studies suggest that incipient neuroinflammation to the early Aβ deposition may be responsible for them.^11^ But other hypotheses point at other mechanisms such as brain swelling, increased neural activity or neural hypertrophy.^12–14^

In this work, we tried to describe and understand the behaviour of these two well-established imaging biomarkers of neurodegeneration: GM volume and brain metabolism ([^18^F]FDG uptake) at the earliest stages of the Alzheimer’s *continuum*. To this aim, we first compared the GM volume and the [^18^F]FDG uptake between Aβ and tau (AT)^15^ groups in a cohort of cognitively unimpaired participants. Then, we analyzed associations between the two imaging modalities and a set of CSF biomarkers measured using the Roche NeuroToolKit which are known to be altered in Alzheimer’s disease and other neurodegenerative diseases, namely markers of amyloid-β (CSF Aβ42/40) and tau pathology (p-tau and t-tau), synaptic dysfunction (neurogranin), neuronal and axonal injury (NfL), glial activation [sTREM2, YKL40, GFAP, cytokine interleukin-6 (IL-6) and S100 calcium-binding protein B (S100b)] and α-synuclein. As some of these biomarkers showed a high degree of collinearity, we clustered them into components to decompose the independent effects of these biomarkers on brain structure and function to better understand the biological underpinnings of cerebral changes in preclinical Alzheimer’s disease stages.

## Materials and methods

### Participants

Participants of this study were part of the ALFA+ cohort, nested in the ALFA (for Alzheimer’s and FAmilies) parent cohort.^16^ The ALFA cohort was established as a research platform to characterize preclinical Alzheimer’s disease in 2743 cognitively unimpaired individuals, aged between 45 and 75 years old, and enriched for family history of sporadic Alzheimer’s disease. ALFA+ participants were selected for a more comprehensive evaluation including clinical and cognitive assessments, a lumbar puncture and an Aβ and [^18^F]FDG PET. All ALFA+ participants were cognitively unimpaired with a Mini-Mental State Examination above 26 and a Clinical Dementia Rating of 0 and were enriched for *APOE-ɛ4* allele and family history of Alzheimer’s disease. For this study, we included the first 319 consecutive participants that had usable CSF and Aβ PET data acquired in <1 year. We also calculated a modified version of the preclinical Alzheimer’s cognitive composite (PACC, see Supplementary material).

The ALFA+ study (ALFA-FPM-0311) was approved by the Independent Ethics Committee ‘Parc de Salut Mar’, Barcelona, and registered at Clinicaltrials.gov (Identifier: NCT02485730). All participants signed the study’s informed consent form that had also been approved by the Independent Ethics Committee ‘Parc de Salut Mar’, Barcelona. The experiments conformed to the principles set out in the WMA Declaration of Helsinki and the Department of Health and Human Services Belmont Report.

### CSF sampling

CSF levels of Aβ42,^17^ as well as Total-Tau and Phospho-Tau(181P)^18^ were measured using Elecsys® electrochemiluminescence immunoassays on a fully automated cobas e 601 instrument (Roche Diagnostics International Ltd., Rotkreuz, Switzerland). The other CSF biomarkers (Aβ40, NfL, neurogranin, YKL-40, GFAP, sTREM2, S100b, IL-6 and α-synuclein) were measured with robust prototype assays as part of the Roche NeuroToolKit on cobas e 411 and e 601 instruments (Roche Diagnostics International Ltd., Rotkreuz, Switzerland). All available participants with CSF biomarkers were used in this study. All measurements were performed at the Clinical Neurochemistry Laboratory, Sahlgrenska University Hospital, Mölndal, Sweden, by board-certified laboratory technicians who were blinded to diagnostic and other clinical data.

Individuals were then classified by AT groups using very sensitive cut-offs of CSF Aβ42/40 ratio (A+: <0.071) and p-tau (T+: >24 pg/mL), previously validated for research purposes.^2^ A − T+ participants (*n* = 12) were excluded from further analyses as they are thought to reflect non-Alzheimer’s disease pathological changes.^1^ The extreme values of each CSF biomarker, defined as those that fell outside three times the interquartile range above the third quartile or below the first quartile, were also removed.

CSF biomarkers were then grouped into three main categories: core Alzheimer’s disease biomarkers, glial biomarkers and markers of neurodegeneration. Core Alzheimer’s disease biomarkers were related to Aβ and tau pathophysiology and included Aβ42/40 ratio and p-tau and t-tau, respectively. Glial biomarkers were those related to micro- or astroglial-related pathways and included: GFAP, YKL-40, sTREM2, s100b and IL-6. Finally, NfL and α-synuclein were considered neurodegeneration markers and neurogranin a synaptic dysfunction marker.

### Image acquisition

All participants had a T1-weighted MRI scan, a [^18^F]FDG PET scan and a [^18^F]flutemetamol PET scan acquired within 1 year of the CSF determinations (82 ± 181 days). A high-resolution 3D T1-weighted MRI sequence was acquired in a 3 T Philips Ingenia CX scanner (TE/TR = 4.6/9.9 ms, Flip Angle = 8°; voxel size = 0.75 × 0.75 × 0.75 mm^3^). PET scans were acquired in a Siemens Biograph mCT scanner. [^18^F]FDG PET scans were acquired 45 min after the administration of 185 MBq of [^18^F]FDG and images were reconstructed using a OSEM3D algorithm (8 iterations, 21 subsets) with PSF + TOF corrections. Centiloid (CL) values ^19^ were obtained from [^18^F]flutemetamol PET scans to characterize cerebral Aβ deposition in the participants, as described in a previous study.^20^

### Image processing

Modulated GM volume maps were obtained using the DARTEL implementation for voxel-based morphometry in SPM12. These images were finally smoothed with a three-dimensional Gaussian kernel of 12 mm full width at half maximum (FWHM).

[^18^F]FDG PET scans were co-registered to the corresponding T1-weighted MRI scans at the T1 space and normalized to the standard MNI space using the transformation calculated for the MRI scan using the DARTEL implementation. [^18^F]FDG uptake in the brain was normalized to the cerebellar vermis.^21^ Spatially normalized [^18^F]FDG scans were finally smoothed by a three-dimensional Gaussian kernel of 12 mm FWHM.

### Statistical analyses

#### Demographics

Differences in demographics by AT groups were assessed using one-way ANOVA or χ^2^ test depending on the nature of the variable.

#### Structural and metabolic differences by AT groups

The first objective of our study was to investigate differences in two different imaging markers of neurodegeneration by AT groups in a cohort of cognitively unimpaired individuals. To this aim, we performed independent one-way ANCOVA for the two sets of images. A voxel-wise analysis was performed with modulated T1-weighted images and [^18^F]FDG PET scans, in two independent models, set as the dependent variable and AT grouping (*i.e.* A − T-, A + T- and A + T+) as the independent variable coded with dummy regressors. Age, sex and *APOE-ɛ4* status (*i.e.* carriers/non-carriers) were used as covariates in both sets of analyses and total intracranial volume (TIV) was also included as covariate for the T1-weighted analysis. Contrasts were designed to test all pairwise comparisons in both directions.

#### Structural and metabolic associations with raw CSF biomarkers

As per our second main objective, we assessed individual associations between CSF biomarkers and each of the two imaging modalities. We used multiple regression models in which images were used as dependent variable and CSF biomarkers as independent variables. We created independent models per each CSF biomarker and image modality. Age, sex, *APOE-ɛ4* carriership and TIV (only for T1-weighted images) were used as covariates. Positive and negative associations were tested in all cases.

#### Combination of CSF biomarkers

Given the high similarity between the associations between some of the CSF biomarkers and the images, we calculated the cross-correlation between all CSF biomarkers using Pearson’s r. As we observed high cross-correlations among some of the analyzed CSF biomarkers (see Supplementary Fig. 1), in a second step we chose to address the associations with the neuroimaging variables using a non-negative matrix factorization (NNMF). With this approach we aim at determining a combination of CSF biomarkers that better explained the variability observed in the images.

In this study, we used NNMF to cluster CSF biomarkers into a few components. NNMF is similar to principal component analysis (PCA) as it is used to ‘explain the observed data using a limited number of basis components, which when combined together approximate the original data as accurately as possible.^22^ Mathematically, NNMF decomposes an input matrix (original data) into a component matrix (combination of CSF biomarkers) and a weight matrix (loadings of CSF biomarkers). An important aspect of this methodology is that all three matrices used (*i.e.* original data, component, and weight matrices) have non-negative values. Therefore, the final result of this decomposition will be a set of components formed by the weighted sum of all CSF biomarkers. These weights can be higher or lower but will always be positive. Furthermore, these components do not need to be orthogonal nor depend on the other components. These two characteristics are important aspects as they facilitate the interpretation of the results.

We used the *NMF* package from R to perform the NNMF.^22^ Before performing the NNMF we normalized the data in a range from 0 to 1 to be able to compare the loadings of the components. Of note, all biomarkers were normalized as the maximum value being linearly transformed to 1 and the minimum to 0, except the Aβ42/40 ratio in which we reversed the transformation so higher values represented higher Aβ pathology. These normalized values were the input of the NNMF. Given that this technique removes the whole row (subject) of the data if one or more columns (CSF biomarkers) are not available, we imputed the data to avoid losing information (see Supplementary material). The number of components (*i.e.* combination of CSF biomarkers), *r* = 3, was selected as the smallest for which the marginal decrease in the residuals remains larger than the decrease observed for randomized data (See Supplementary material for further details).^23^

To better characterize each component, we also investigated its expression as a function of AT stages and basic demographic and genetic characteristics (age, sex and *APOE-ɛ4* status). For categorical variables we conducted independent ANCOVA to test differences in components’ expression by groups. Age, sex and *APOE-ɛ4* carriership were used as covariates appropriately. A linear regression was used to test associations between components’ expression and age, using sex and *APOE-ɛ4* carriership as covariates. Additional analyses adding education as covariate were conducted with no significant changes observed. Therefore, we hereby present only non-education adjusted results.

#### Structural and metabolic associations with combinations of CSF biomarkers

Once the components from the NNMF were constructed, we used them for our second main objective that was looking at associations between the available CSF biomarkers (NNMF components) and the imaging markers of neurodegeneration. We performed these analyses at the voxel level using independent regression analyses for the two modalities. The images of each modality were used as dependent variables in two different models, and all components were included in each model as independent variables. Age, sex and *APOE-ɛ4* status (and TIV for T1-weighted scans) were used as covariates.

As an additional analysis, we also analyzed the association between the CSF components and a measure of global cognition (PACC) adding education as a covariate in the models.

Analyses were performed with R (v.4.1.0.) and SPM12. For imaging analyses, two levels of statistical significance were set, one more liberal (*P* < 0.005 uncorrected for multiple comparisons) and another more conservative (*P* < 0.001 uncorrected for multiple comparisons), both with a minimum cluster size of *k* > 100 voxels. For the rest of analyses two-sided *P* < 0.05 uncorrected for multiple comparisons was set as the statistical significance threshold.

### Data availability

The data that support the findings of this study are available from the corresponding authors, upon reasonable request.

## Results

The group of participants with valid CSF biomarkers and available imaging biomarkers of neurodegeneration were 319 (A − T-: 205, A + T-: 89 and A + T+: 25). Participants’ characteristics are described in Table 1 by AT groups. Mean (SD) age of the sample was 61.1 (4.7) years old, with A + T+ being significantly older than the other two groups. There was a higher proportion of women [*n* = 199 (62.4%)] with no differences across groups and 173 (54.2%) *APOE-ɛ4* carriers with A-T- group having a significant lower proportion of carriers than the other AT groups.

**Table 1:** Demographics by AT stages. Mean and standard deviations are shown unless otherwise stated

	All (*n* = 319)	A−T− (*n* = 205)	A+T− (*n* = 89)	A+T+ (*n* = 25)
Age, years	61.1 (4.7) [50.0 − 73.6]	60.5 (4.3) [50.0−69.8]	61.7 (5.1) [50.1−70.2]	64.1 (4.6) [51.8−73.6]
Women, n(%)	199 (62.4)	133 (64.9)	49 (55.1)	17 (68.0)
*APOE-ε4* carriers, n(%)	173 (54.2)	85 (41.5)	73 (82.0)	15 (60.0)
Education, years	13.4 (3.5)	13.5 (3.5)	13.3 (3.5)	12.0 (3.5)
Centiloids	3.0 (17.0)	−4.6 (6.5)	12.7 (17.3)	30.6 (27.3)
CSF biomarkers				
Aβ42/40 ratio	0.074 (0.019)	0.087 (0.009)	0.054 (0.011)	0.044 (0.012)
p-tau	15.6 (5.9)	13.9 (4.2)	15.5 (4.1)	29.6 (4.3)
t-tau	193 (64)	176 (49)	191 (44)	340 (52)
Neurogranin	777 (298)	722 (252)	740 (220)	1360 (281)
NfL	81 (26)	75 (24)	83 (23)	117 (29)
GFAP	7.5 (2.3)	7.1 (2.1)	7.5 (2.2)	10.3 (2.2)
YKL-40	145 (51)	137 (45)	143 (44)	215 (64)
sTREM2	7.8 (2.1)	7.6 (1.9)	7.6 (2.0)	10.1 (2.7)
S100b	1.01 (0.22)	0.98 (0.20)	1.04 (0.24)	1.14 (0.29)
IL-6	3.8 (1.2)	3.8 (1.1)	3.7 (1.3)	4.1 (1.5)
α-synuclein	191 (68)	182 (64)	179 (50)	301 (62)
Cognition				
PACC, z-score	0.01 (0.67)	0.01 (0.65)	0.10 (0.68)	−0.31 (0.71)

Abbreviations: Aβ = amyloid-β; GFAP = glial fibrillary acidic protein; IL-6 = cytokine interleukin-6; NfL = neurofilament light; PACC, preclinical Alzheimer's cognitive composite; p-tau = phosphorylated tau; S100b = S100 calcium binding protein B; sTREM2 = soluble triggering receptor on myeloid cells 2; SUVr, standardized uptake value ratio; t-tau = total tau.

### Structural and metabolic differences by AT groups

**Figure 1:**
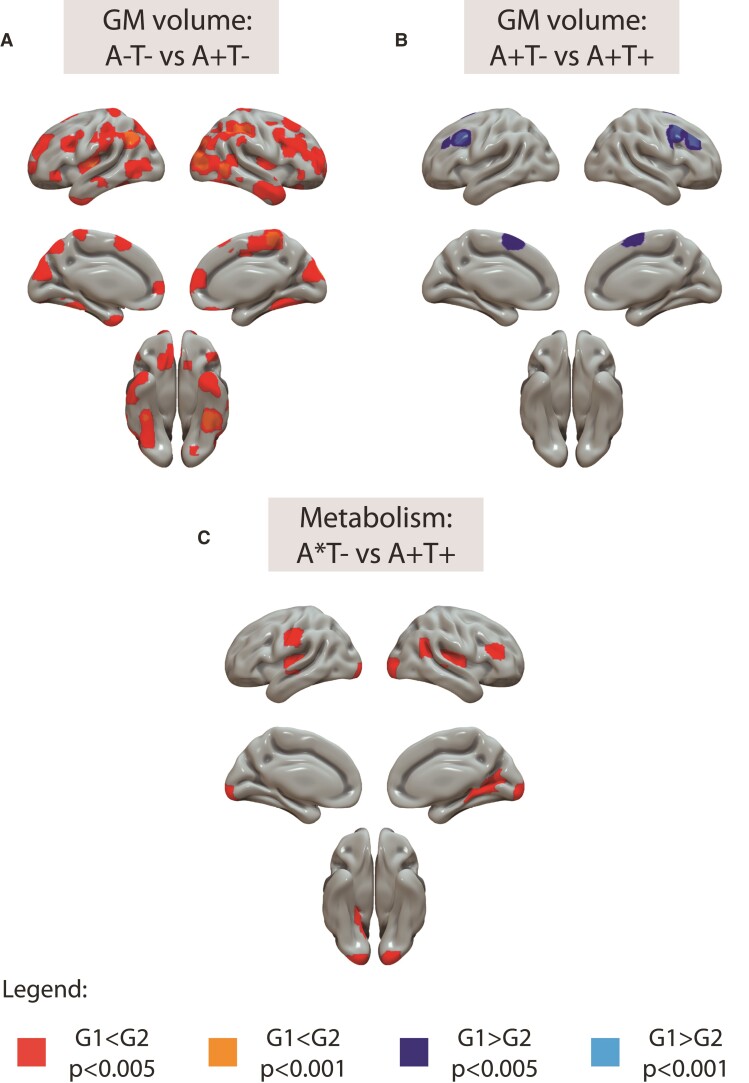
**Structural and metabolic differences by AT stages.** Differences are shown at the voxel level. T1-weighted (A and B) or [^18^F]FDG (C) scans were used as dependent variables in independent models using AT stages as independent variables and age, sex and *APOE-ɛ4* carriership as covariates. Differences in which group 2 (G2) is higher than group 1 (G1) are shown in warm colours (red: *P* < 0.005, orange: *P* < 0.001), whereas the reverse is shown in cold colours (dark blue: *P* < 0.005, light blue: *P* < 0001). A*T- include both A-T- and A*T- groups. GM = grey matter.

Differences in the imaging markers of neurodegeneration by AT groups are depicted in Fig. 1. GM volumes were larger in A + T- individuals compared with the reference (A-T-) in bilateral inferior and middle temporal, insula, cuneus and parietal areas. No differences were observed in [^18^F]FDG uptake between these two groups. In the next groups’ comparison, A + T+ individuals had smaller GM volumes in bilateral frontal gyrus than A + T- individuals. On the other hand, higher [^18^F]FDG uptake was observed in the bilateral transverse temporal gyrus, right calcarine and bilateral occipital pole in A + T+ compared with A*T- group (*i.e.* Aβ-negative tau-negative and Aβ-positive tau-negative).

#### Structural and metabolic associations with raw CSF biomarkers

**Figure 2:**
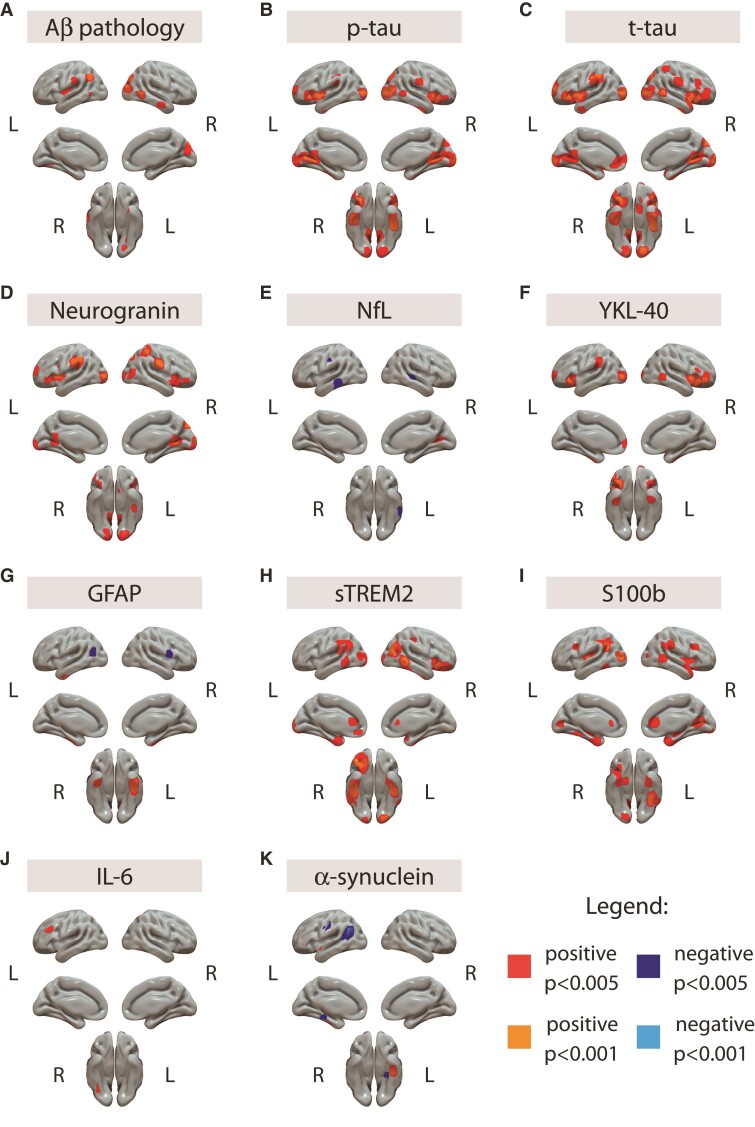
**Association between raw CSF biomarkers and GM volumes.** T1-weighted scans were used as dependent variables in independent models using each CSF biomarker as independent variable and age, sex and *APOE-ɛ4* carriership as covariates. Positive associations are shown in warm colours (red: *P* < 0.005, orange: *P* < 0.001) and negative associations are shown in cold colours (dark blue: *P* < 0.005, light blue: *P* < 0001). Of note, CSF Aβ42/40 levels were inverted with the aim that higher levels would represent higher Aβ pathology, therefore positive associations mean increases in GM volumes with higher Aβ pathology (lower Aβ42/40 levels). Aβ = amyloid-β; GFAP = glial fibrillary acidic protein; GM = gray matter; IL-6 = cytokine interleukin-6; NfL = neurofilament light; p-tau = phosphorylated tau; S100b = S100 calcium binding protein B; sTREM2 = soluble triggering receptor on myeloid cells 2; t-tau = total tau.

**Figure 3:**
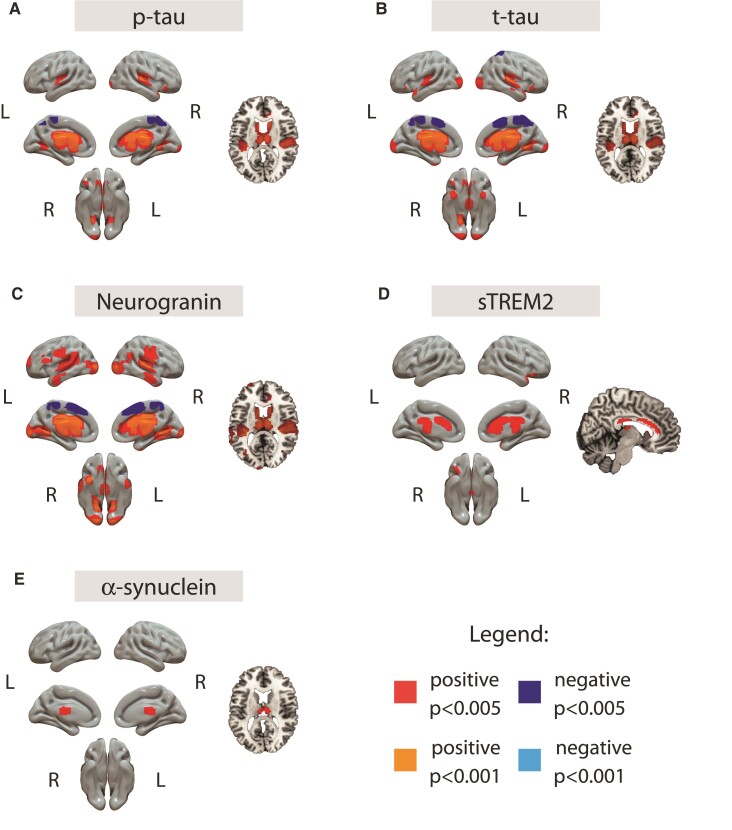
**Association between raw CSF biomarkers and metabolism.** [^18^F]FDG scans were used as dependent variables in independent models using each CSF biomarker as independent variable and age, sex and *APOE-ɛ4* carriership as covariates. Biomarkers not shown in the figure did not present any significance association with brain metabolism. Positive associations are shown in warm colours (red: *P* < 0.005, orange: *P* < 0.001) and negative associations are shown in cold colours (dark blue: *P* < 0.005, light blue: *P* < 0001). GM = grey matter; p-tau = phosphorylated tau; sTREM2 = soluble triggering receptor on myeloid cells 2; t-tau = total tau.

Structural and metabolic associations with raw CSF biomarkers are shown in Figs 2 and 3, respectively. Briefly, we observed positive associations between GM volume and CSF levels of p-tau, t-tau, neurogranin, YKL-40, sTREM2 and S100b in similar frontal and temporal areas. Aβ pathology also showed a positive association with GM volume in temporo-occipital areas. Although less extended, NfL presented negative associations with GM volume in bilateral inferior temporal cortices. GFAP and α-synuclein showed negative associations in a temporo-occipital cluster and positive in bilateral temporal pole. Finally, IL-6 showed minimal positive associations with GM volume.

A similar pattern was observed for p-tau, t-tau and neurogranin with positive association with [^18^F]FDG uptake associations in bilateral transverse temporal gyrus and striatum, but negative associations in medial frontal/parietal regions of the brain. The other two main CSF biomarker’s levels showing significant positive associations with [^18^F]FDG uptake were sTREM2, mainly in anterior and posterior cingulate gyri and thalamus and α-synuclein in the thalamus.

#### Structural and metabolic associations with combinations of CSF biomarkers

**Figure 4:**
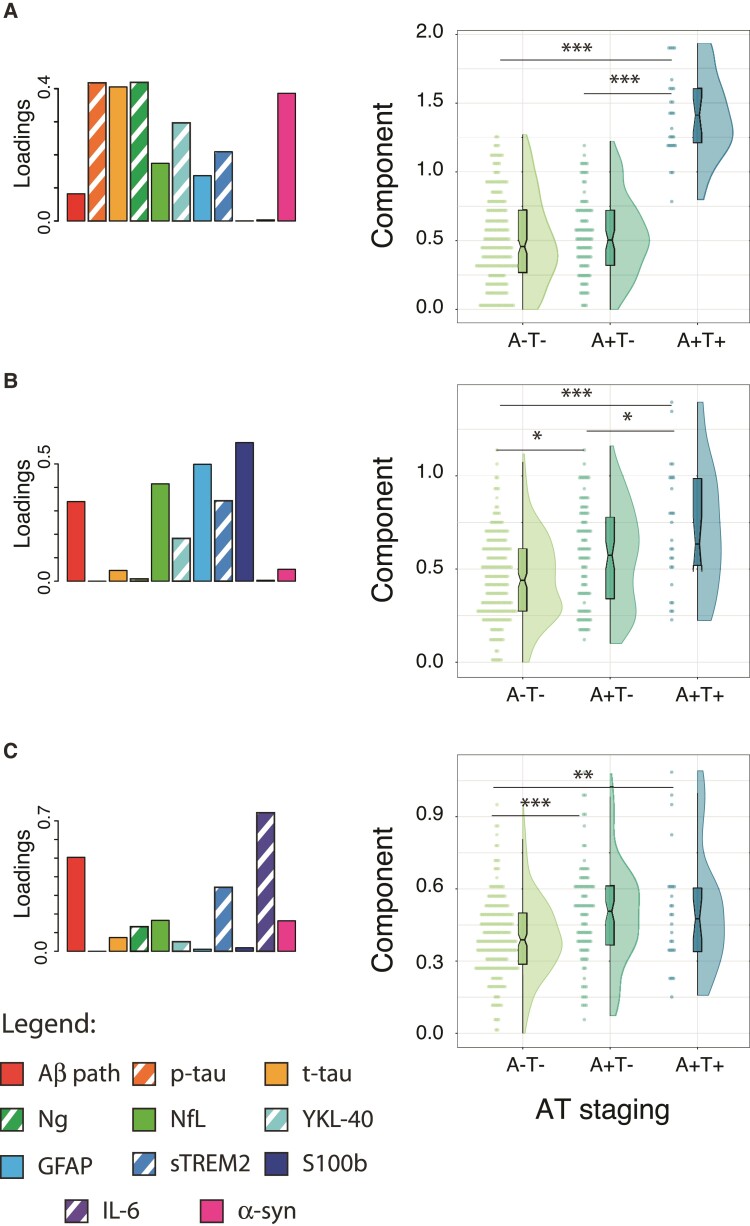
**CSF components’ characterization by AT stages.** The description of components’ loadings is shown in the first column and expression of each component by AT stages in the second. Significant differences are depicted in the figure. Of note, CSF Aβ42/40 levels were inverted with the aim that higher expression would represent higher Aβ pathology. * *P* < 0.05; ** *P* < 0.01; ***P < 0.001. Aβ = amyloid-β; GFAP = glial fibrillary acidic protein; GM = gray matter; IL-6 = cytokine interleukin-6; NfL = neurofilament light; Ng = neurogranin; p-tau = phosphorylated tau; S100b = S100 calcium binding protein B; sTREM2 = soluble triggering receptor on myeloid cells 2; t-tau = total tau.

**Figure 5:**
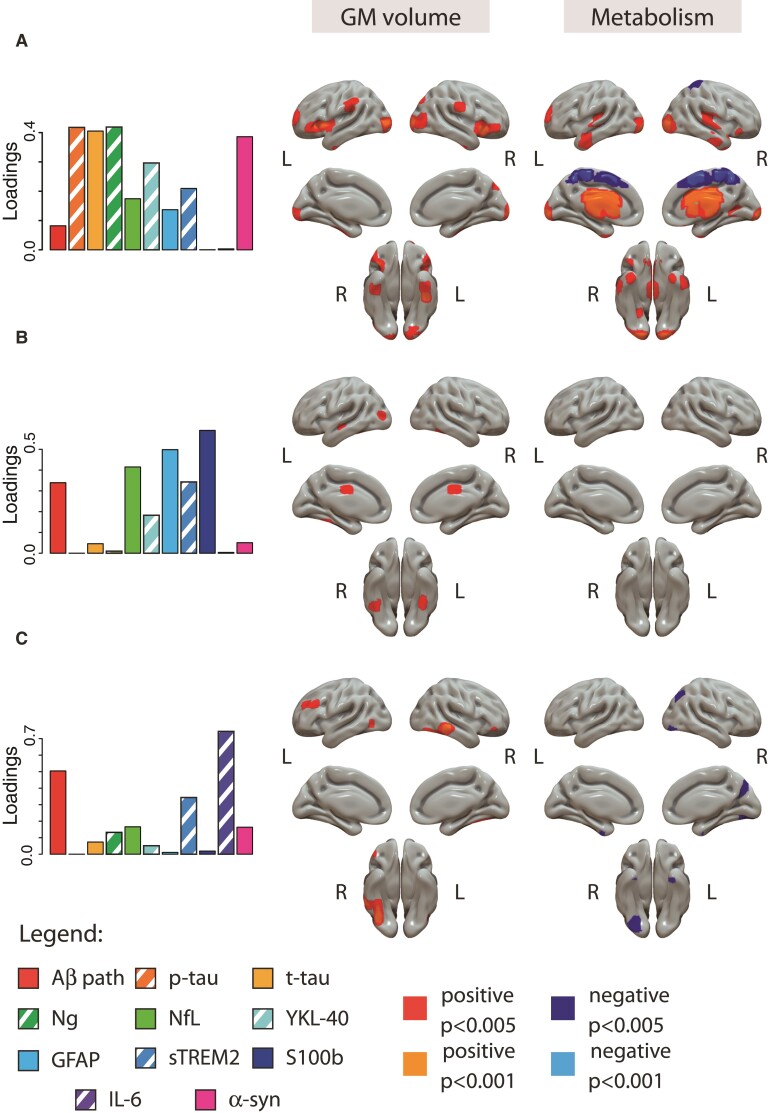
**Association between CSF components and imaging markers.** Associations at the voxel level are shown for structure (second column) and glucose metabolism (third column). Component's weights are depicted in the first column for reference. Positive associations are shown in warm colours (red: *P* < 0.005, orange: *P* < 0.001) and negative associations are shown in cold colours (dark blue: *P* < 0.005, light blue: *P* < 0001). Of note, CSF Aβ42/40 levels were inverted with the aim that higher expression would represent higher Aβ pathology. T1-weighted or [^18^F]FDG scans were used as dependent variables in independent models using all the components as independent variables in the same model and age, sex and *APOE-ɛ4* carriership as covariates. Aβ = amyloid-β; GFAP = glial fibrillary acidic protein; GM = gray matter; IL-6 = cytokine interleukin-6; NfL = neurofilament light; Ng = neurogranin; p-tau = phosphorylated tau; S100b = S100 calcium binding protein B; sTREM2 = soluble triggering receptor on myeloid cells 2; t-tau = total tau.

Three components were constructed using the NNMF approach, with all CSF biomarkers included in all of them. They were differently associated with AT staging as can be observed in Fig. 4. Both structural and metabolic associations with these components are shown in Fig. 5.

The first component was formed by biomarkers related to tau pathology (p-tau and t-tau), neurodegeneration (mainly driven by neurogranin and α-synuclein but with also a smaller proportion of NfL), and glial activation (mainly the astroglial marker YKL-40 and sTREM2, with also smaller involvement of GFAP). This component’s expression was significantly increased in A+T+ participants compared to the other two A*T- groups (*i.e.* Aβ-negative tau-negative and Aβ-positive tau-negative). On its relation to the neuroimaging variables, this component showed a positive association with GM volumes (*i.e.* higher values of these CSF biomarkers were related with larger GM volumes) in bilateral insula, bilateral inferior temporal, bilateral supramarginal and inferior occipital cortices. With metabolism, it showed a strong positive association with bilateral thalamus and striatum, and also some positive associations in the inferior temporal and transverse temporal gyri. This component also presented negative associations with metabolism in superior frontal/parietal medial areas.

The second component was mainly driven by glial biomarkers, especially S100b, and Aβ pathology with a significant contribution of NfL, a neurodegeneration marker. We observed a monotonic increase of this component’s expression by AT stages. This component showed a significant although small positive association with GM volumes in bilateral middle cingulate gyrus and inferior temporal cortices. No significant association was observed between this component and metabolism.

Finally, the third component was driven by IL-6 levels, although it also had an important contribution from Aβ pathology and another glial marker, sTREM2. Here, component’s expression was elevated in A+T* groups (*i.e.* Aβ-positive tau-negative and Aβ-positive tau-positive). This component was mainly associated with higher GM volumes in the inferior right temporal cortex. Regarding its association with metabolism, it showed a negative relationship with metabolism in bilateral caudate (not observed in the figure), inferior temporal cortices and right precuneus.

The specific weights of each CSF biomarker for each component as well as the components’ expression as a function of basic demographic characteristics are reported in Supplementary Tables 1–4 and Figs 2–4.

Regarding cognition (PACC), we only observed an association between higher expression of the second component with worse cognitive performance, only at a trend level [*β_std_(95%CI)* = −0.09 (−0.18,0.00), *P* = 0.089; Supplementay Fig. 5 and Supplementary Table 4].

## Discussion

In this work, we assessed, in cognitively unimpaired individuals, the associations between CSF biomarkers of several pathophysiological mechanisms of neurodegenerative diseases and GM volume and cerebral glucose consumption ([^18^F]FDG uptake). Using an unbiased approach, we showed distinct patterns of pathophysiological markers associated with increased GM volume and [^18^F]FDG uptake in preclinical Alzheimer’s disease stages.

### Structural and metabolic differences by AT groups

We first focused our attention on specific biomarkers of Alzheimer’s disease pathology and found differences in brain structure and metabolism across AT stages,^15^ even in our population of cognitively unimpaired individuals. Structurally, we found higher GM volume in A+T- compared with A-T- subjects but not in the A+T+ group. Regarding metabolic differences, we also observed increases of [^18^F]FDG uptake in A+T+ compared with the other two tau-negative (A*T-) groups. Albeit such these structural and metabolic increments might seem paradoxical since decrements have been typically described in association with Alzheimer’s disease progression and neurodegeneration in *symptomatic* stages, they are in line with numerous previous studies which have observed increased GM volumes/cortical thickness and/or [^18^F]FDG uptake in *preclinical* Alzheimer’s disease stages.^4–9,24^ The fact that we are observing these increments on structure and metabolism in different AT stages may be related to the several biological mechanisms other than neurodegeneration that might be independently driving the observed effects on brain structure and function (see below). We hypothesize that these behaviours may be decoupled at these early preclinical stages and, synchronize in later stages of the disease when neurodegeneration is driving the changes. However, this needs further validation in independent cohorts.

The pattern of increased GM volume found in this study was scattered across the whole brain. Some of the more prominent areas with larger GM volumes in A+T- participants were temporal and temporo-parietal areas, bilateral cuneus, bilateral putamen, and bilateral angular gyrus, but we also found some significant clusters in the occipital and frontal lobe. Some of these areas have been described before as having larger volumes in relationship to Aβ pathology, such as the temporal lobe,^5,7,24^ temporo-parietal areas,^4,8,24^ putamen^6^ and occipital lobe.^6^ Regarding metabolism increases, we observed fewer areas with increments, involving mainly bilateral transverse temporal gyrus, right calcarine and bilateral occipital pole. Studies comparing cerebral glucose metabolism in these early stages are scarce, however, the increments found in our study in the temporal lobe are in agreement with a previous study.^4^ In this study from Johnson et al., the authors showed that from a group of cognitively unimpaired participants, those with higher levels of Aβ pathology showed higher glucose metabolism in similar temporal areas and bilateral thalamus compared with Aβ negative participants. Suggesting that, although paradoxical, our results concur with those found in previous studies.

It must be noted, that despite the resemblance of some of the results, our study has significant differences with respect to many of the aforementioned studies. Given that our cohort comes from a research perspective, our participants differ in important characteristics from previous studies, which mainly come from clinical cohorts. In particular, our subjects are significantly younger (mean age = 61.1 years old) and have higher *APOE-ɛ4* carriership and family history of Alzheimer’s disease prevalence. Thus, our participants may be at earlier stages of the disease, if so, although having a higher risk for Alzheimer’s disease due to their genetic risk factors. It is also important to note, that our sample size is significantly larger than most of those in the previous studies, which may have allowed us to be more sensitive to detect significant differences. Finally, we used a group comparison approach as some of previous studies,^6,24^ based on the extensively used AT staging.^15^ This allowed us to consider the two main pathological hallmarks of Alzheimer’s disease, given that previous studies have suggested synergistic effects on brain structure.^24,25^

We hypothesize that the increments in GM volumes and glucose metabolism are transient changes before the expected neurodegeneration in more advanced stages of the disease. Supporting this idea, previous longitudinal studies have also found significant pathology-related atrophy only in later stages. For instance, a longitudinal study by Desikan and colleagues showed that only subjects that had both Aβ and tau pathology, but not those with only Aβ, presented atrophy in the entorhinal cortex.^25^ Similarly, another longitudinal study showed a decrease in the rate of atrophy, likely age-related, in those subjects that were only Aβ positive compared to biomarker negative subjects. As expected, though, atrophy rates significantly increased in subjects more advanced in the disease stage, when both Aβ and tau were positive.^9^ Altogether, these results support an inverted U-shape for structural changes in Alzheimer’s disease, as previously suggested,^8,9,24^ with a slight GM thickening in early stages, mainly related to Aβ positivity, that will be followed by a pathology-related atrophy process when subjects become tau-positive.

### Structural and metabolic associations with CSF biomarkers

Once we had established the differences in the imaging variables by AT stages, we used the set of CSF biomarkers to better understand the biological mechanisms underlying these structural and metabolic alterations. CSF markers were strongly correlated among them and resulted in similar patterns of associations with the imaging biomarkers. In particular, many of the glial and neurodegeneration markers showed similar associations than those of the tau pathology markers (p-tau and t-tau). Therefore, a direct correlation analysis did not allow us to disentangle the specific effects associated with different pathophysiological mechanisms. To address this, we followed a clustering approach using non-matrix factorization (NNMF), which has allowed us to discover several combinations of CSF biomarkers (*i.e.* components) that are expressed together.

The first component of our factorization explained most of the variability observed in the imaging scans. This component was composed of biomarkers that reflect the primary pathology (CSF p-tau, t-tau and, to a lower extent, Aβ pathology), glial markers (especially YKL-40 and sTREM2), neurogranin and α-synuclein (neurodegeneration-related markers). Higher values of this component were associated with increased [^18^F]FDG uptake in the bilateral thalamus and inferior and transverse temporal gyri, as well as increased GM volume in the bilateral anterior insulae, inferior temporal/fusiform gyri as well as supramarginal gyri. These patterns of structural and metabolic increments closely resemble previous findings by Johnson *et al.*,^4^ who found increased [^18^F]FDG uptake in the bilateral thalamus and temporal cortex in cognitively unimpaired individuals with intermediate and high PIB PET uptake. In this study, no tau marker was available, but it is expected that subjects with the highest PIB PET values had increased tau levels as compared with Aβ-negative subjects. Remarkably, positivity in Aβ PET using typical thresholds is expected to be observed later than with CSF Aβ biomarkers.^26^ Also in line with our findings, individuals in Johnson’s study with high PIB uptake also showed increased GM volume in the lateral parietal and ventral temporal lobes, albeit not surviving correction for multiple comparisons.

The novelty in our study is that we can link this pattern of higher GM volume and [^18^F]FDG uptake with other pathophysiological mechanisms using different CSF biomarkers. In this regard, this pattern was observed in association with increased YKL-40 levels and, to a lower extent, sTREM2. YKL-40 seemed more specifically expressed in association with p-tau in this first component as sTREM2 was similarly expressed in this and the rest of the components. In the Alzheimer’s disease *continuum*, higher levels of CSF YKL-40, a marker of reactive astrogliosis, have been frequently described with increased levels of p-tau^27^ and in associations with increased GM volume and cortical thickness in preclinical Alzheimer’s disease stages.^28,29^ Microglial activation, here represented by higher sTREM2 levels, has also been related to higher GM volumes in early stages of Alzheimer’s disease.^11^ Here, we suggest that all these pathophysiological mechanisms may be more tightly associated than previously thought. Overall, results with this component suggest that astrocytic and microglial reactivity related with early tau pathology underlies the observed pattern of increased GM volume and hypermetabolism. However, our experimental approach cannot decipher the underlying inflammatory mechanisms underlying these associations. Previous reports observing increments in these early phases of Alzheimer’s disease have suggested multiple non-exclusive biological explanations such as cellular hypertrophy,^5,8,24^ oedema or brain swallowing^5^ and the buildup of Aβ plaques.^30^ On this regard, neuropathological studies have provided some proofs of neuronal hypertrophy in close relationship with Aβ pathology.^13^ Future studies trying to further investigate these effects may benefit from including other imaging techniques such as T1/T2 relaxometry for looking at oedema or other cortical fluid/myelin changes,^31^ or neurite orientation dispersion and density imaging to investigate microstructural aspects of axons and dendrites^32^ using MRI. PET tracers targeting microglial and astroglial activation, like those binding to translocator protein 18 kDa (TSPO) or monoamine oxidase B (MAO-B), may also help us to decipher these underlying mechanisms in the near future.^33^ Finally another hypothesis that cannot be disregarded is that these differences might be related to brain reserve,^34^ which may have helped these subjects to maintain their cognitive abilities although their pathology.^5^

Contrary to the first component in which the levels of glial markers seem to be tightly related to tau pathology, the second component had a significant contribution from glial and neurodegeneration (NfL) markers together with Aβ, but not tau, pathology. This may indicate a different relationship between glial markers and the two neuropathological hallmarks of Alzheimer’s disease. It is important to note, though, that the contribution from glial markers on these two first components was substantially different. For instance, in this second component S100b and GFAP, both astroglial-related markers,^35^ had the highest weights in comparison to in the first component which was maily driven by YKL-40 and sTREM2. This translated to different associations with brain structure and metabolism. In particular, we observed significant positive associations in bilateral middle cingulate and middle temporal gyri. Although not reaching the stricter statistical threshold, the symmetrical pattern of these associations advocates for a true, albeit rather small, significant effect. Regarding its expression along the preclinical Alzheimer’s disease *continuum*, this component increased monotonically with progressive AT stages, in comparison with the first component that was only elevated in A+T+ participants. These results suggest that these markers of astrogliosis, CSF GFAP and S100b are progressively expressed with accumulating Aβ pathology and that they track a different aspect of astroglial response to Alzheimer’s disease pathology than YKL-40, which was only found to be elevated in the A + T+ stage.

Another glial-related marker, the inflammatory cytokine interleukin-6 (IL-6), mostly contributed to the third component, together with increased Aβ pathology and elevated sTREM2 levels. Higher values of this component were associated with greater GM volume in bilateral middle temporal gyri (although it only reached the more robust threshold in the right hemisphere) and cerebellum. The negative association between this component and metabolism was restricted to small clusters in bilateral temporal pole and right precuneus. Of note, IL-6 has not been found elevated with Alzheimer’s disease pathophysiology in our sample, unlike the rest of the CSF biomarkers measured in this study.^2^ This together with the fact that IL-6 showed no effect on other components suggests that this marker may be measuring another independent glial reaction to what was previously found in the other two components, and in this case, more directly related to the presence of very early Aβ pathology. In support of this hypothesis, this component was found to be elevated in the two A+ groups. Of note, unlike the other two components, this third one was significantly more expressed in *APOE-ɛ4* carriers and did not show an association with age. In cognitively unimpaired individuals, the *APOE-ɛ4* allele has been consistently shown to promote earlier and more abundant Aβ pathology.^36^ Besides, it has also been suggested that *APOE-ɛ4* may influence the immunological response to Aβ deposition.^37^ Given the high prevalence of Aβ-positive *APOE-ɛ4* carriers in our sample, this third component might be reflecting an *APOE-ɛ4*-modulated immunological response to early Aβ deposition.

Although not found with our components, we also observed some negative associations between raw neurodegeneration-related markers and GM volumes. In particular, NfL and α-synuclein showed negative associations in temporal and temporo-parietal cortices, respectively. Although the effect of NfL in our study this effect was found in rather limited brain areas, these are among the earliest areas of tau accumulation in Alzheimer’s disease, which is promptly followed by an atrophy process. Such an imaging pattern suggests that this observation may be associated with very early and specific Alzheimer’s disease-related neurodegeneration. In line with our findings, a previous study that reported the associations between CSF NfL and cortical thickness showed a similar pattern of thinning in A+ cognitively unimpaired subjects,^38^ which expanded to additional cortical areas in more advanced stages of the disease. In addition to the agreement, Benedet *et al.*^39^ showed that increased plasma NfL in cognitively unimpaired individuals was associated with a longitudinal decrease of GM volumes in inferior and medial temporal areas. Therefore, the limited effect observed in our sample might be explained by it being mostly formed by participants at the earliest stages of the Alzheimer’s *continuum*, when neurodegeneration is still incipient.

#### Strengths and limitations

In terms of study strengths, we presented here the results of a very well-characterized sample of cognitively unimpaired individuals with both fluid and neuroimaging biomarkers. Of note, these fluid biomarkers were all measured on a single fully automated and precise immunoassay platform. This permitted us to investigate associations at the earliest stages of the Alzheimer’s *continuum* with a particular focus on non-core Alzheimer’s disease biomarkers. Further, the use of NNMF allowed us to disentangle associations with imaging markers of multiple CSF biomarkers despite them being tightly correlated. Here, we studied the associations of CSF components with both structural and metabolic imaging modalities. Although both of these modalities are used rather interchangeably to monitor neurodegeneration in Alzheimer’s disease, our results of the voxel-wise analyses show marked differences between them in their associations with CSF biomarkers in preclinical Alzheimer’s disease stages.

As for limitations, this being a cross-sectional study, it does not allow us to confirm the prognostic value of the discovered components of CSF biomarkers. We found a complex interplay between CSF and imaging biomarkers that suggests non-monotonic trajectories along the early Alzheimer’s disease *continuum*, which can only be confirmed with a longitudinal follow-up of this sample, currently underway. Another limitation of this study is that the studied sample is formed by cognitively unimpaired individuals who are, therefore, expected to show minimal neurodegeneration, at maximum. The lack of a more evident neurodegenerative effect in our sample may limit the variability in the imaging markers of neurodegeneration and lead to a lack of statistical power. This limitation is even more severe given the relatively small number of A+T+ participants in our sample. Nevertheless, we could still detect statistically significant associations between CSF biomarkers and neuroimaging modalities. We acknowledge that we used a rather lenient threshold for significance in the voxel-wise analysis which combined with the high number of comparisons here performed may have increased the risk of false positives. However, we also observe that many of the associations with the raw CSF biomarkers lead to very similar results, lowering the probability of being false-positive results. Nonetheless, to try to minimize this we used two different thresholds to show both a more liberal and a more stringent significance. We acknowledge that even our stricter threshold could be thought of as liberal. In this regard, we consider this a hypothesis-generating study. Effects here described needing to be confirmed in independent samples covering the whole Alzheimer’s disease spectrum and, again, with longitudinal follow-up.

## Conclusions

To conclude, we showed higher GM volumes and [^18^F]FDG uptake in preclinical Alzheimer’s disease, which are mainly associated with increased levels of CSF glial biomarkers in association with altered Alzheimer’s disease pathology biomarkers. Our results suggest that distinct mechanisms trigger glial activation at different preclinical Alzheimer’s disease stages in response to Aβ or tau pathology, which may lead to transient increments in imaging markers considered to reflect neurodegeneration. Such increments appear to revert at later stages of disease in association with higher levels of CSF NfL, even in preclinical stages. Our results may help to better understand the interrelationship among biomarkers of core Alzheimer’s disease pathology and those of associated pathophysiological mechanisms that are already altered in the earliest stages of Alzheimer’s disease, which can inform the design of clinical trials of preventive therapies targeting these mechanisms.

## Supplementary Material

fcac134_Supplementary_Data

## Appendix

Collaborators of the ALFA study are: Annabella Beteta, Anna Brugulat-Serrat, Alba Cañas, Irene Cumplido, Carme Deulofeu, Ruth Dominguez, Maria Emilio, Sherezade Fuentes, José María González-de-Echavarri, Oriol Grau-Rivera, Laura Hernandez, Gema Huesa, Jordi Huguet, Iva Knezevic, Paula Marne, Tania Menchón, Maria Pascual, Albina Polo, Sandra Pradas, Aleix Sala-Vila, Anna Soteras, Laia Tenas, Marc Vilanova and Natalia Vilor-Tejedor.

## Abbreviations

Aβ =: amyloid-β
FDG =: fluorodeoxyglucose
FWHM =: full width at half maximum
GFAP =: glial fibrillary acidic protein
GM =: grey matter
IL-6 =: cytokine interleukin-6
NfL =: neurofilament light
NNMF =: non-negative matrix factorization
PACC =: preclinical Alzheimer’s cognitive composite
p-tau =: phosphorylated tau
ROI =: region of interest
S100b =: S100 calcium-binding protein B
sTREM2 =: soluble triggering receptor on myeloid cells 2
TIV =: total intracranial volume
t-tau =: total tau
