# Supplementary material for: Brain alterations in the early Alzheimer’s continuum with amyloid-β, tau, glial and neurodegeneration CSF markers
Source: Brain Commun. 2022 May 24;4(3):fcac134. doi: 10.1093/braincomms/fcac134 (PMC9185381; doi:10.1093/braincomms/fcac134)
Supplement: fcac134_Supplementary_Data [file fcac134_supplementary_data.docx]

**Brain alterations in the early Alzheimer's *continuum* with amyloid, tau, glial and neurodegeneration CSF markers**

Gemma Salvadó, Mahnaz Shekari, Carles Falcon, Grégory Operto, Marta Milà-Alomà, Gonzalo Sánchez-Benavides, Raffaele Cacciaglia, Eider Arenaza-Urquijo, Aida Niñerola-Baizán, Andrés Perissinotti, Carolina Minguillon, Karine Fauria, Gwendlyn Kollmorgen, Ivonne Suridjan, José Luis Molinuevo, Henrik Zetterberg, Kaj Blennow, Marc Suárez-Calvet, Juan Domingo Gispert for the ALFA Study

## Materials and methods

### PACC calculation

### A Preclinical Alzheimer Cognitive Composite (PACC) like score was calculated based on the one proposed by Donohue *et al,*^1^ and the later proposals made by Papp *et al,*^2^ and Jonaitis *et al.*^3^ According to this previous work we have dropped the Mini-Mental State Examination (MMSE) because of a lack of sensitivity in our sample and have included a categorical fluency measure. The variables included in the composite were: Free and Cued Selective Reminding Test (FCSRT) Total Immediate recall (0-48), Wechsler Memory Scale (WMS) logical memory Total Delayed recall (0-50), Wechsler Adult Intelligence Scale (WAIS) - Coding (0-135), and Semantic Fluency (animals in 1 minute).The procedure to create the PACC was as follows: 1) Standardization of each individual raw scores into z-scores using as reference the means and standard deviations (SDs) obtained from the biomarker negative sample (A-T-N-). 2) Averaging the 4 obtained z-scores into a single score. 3) Re-standardization of the obtained PACC to ensure a distribution with mean=0 and SD=1 using the means and SD from the biomarker negative group as done in Step 1.

### Non-negative matrix factorization

Before performing the normalization of the CSF values, we performed a very basic imputation method to avoid losing subjects. We replaced the missing data with the mean of that CSF biomarker for the AT group of that subject. This way we did not bias the imputation, especially in the subjects in more advanced stages (e.g., A+T+).

To decide the optimal number of components for the non-negative matrix factorization (NNMF), we performed multiple steps. First, we performed the NNMF with different ranks (*r*) ranging from two to eleven (number of the total CSF biomarkers). For each rank, NNMF was estimated as the best of 100 runs with random initializations to avoid finding local minima. As a second step, we repeated this procedure with randomized data. Following Frigyesi’s criteria,^4^ we selected *r* as the smallest factorization rank for which the marginal decrease in the residuals remains larger than the decrease observed for randomized data. Once the optimal rank was selected (*r* = 3), we repeated the NNMF procedure with this rank using 200 runs.

## Tables and figures

**Supplementary Figure 1 Cross-correlation between CSF biomarkers.** Correlation, as measured as Pearsons's r, among raw CSF biomarkers is shown in A. Correlation among components after NNMF estimation is shown in B. We can see in the figure a high correlation among CSF biomarkers before performing the NNMF that almost disappears with the creation of the components.

Abbreviations: Aβ = amyloid-β; GFAP = glial fibrillary acidic protein; IL-6 = cytokine interleukin-6; NfL = neurofilament light; NNMF, non-negative matrix factorization; p-tau = phosphorylated tau; S100b = S100 calcium binding protein B; sTREM2 = soluble triggering receptor on myeloid cells 2; t-tau = total tau.

|  | C1 | C2 | C3 |
| --- | --- | --- | --- |
| Aβ | 9.5 | 32.4 | 48.1 |
| p-tau | 47.4 | 0.0 | 0.0 |
| t-tau | 46.0 | 4.4 | 7.0 |
| Neurogranin | 47.5 | 1.1 | 12.7 |
| NfL | 20.1 | 39.1 | 15.4 |
| YKL-40 | 33.8 | 17.2 | 4.7 |
| GFAP | 16.1 | 46.7 | 0.3 |
| sTREM2 | 24.0 | 32.6 | 32.5 |
| S100b | 0.6 | 55.5 | 0.8 |
| IL-6 | 0.1 | 1.3 | 71.7 |
| α-synuclein | 43.8 | 4.9 | 15.7 |

**Supplementary Table 1 Component's weights.** Actual weights of each CSF component after the NNMF modelling.

Abbreviations: Aβ = amyloid-β; GFAP = glial fibrillary acidic protein; IL-6 = cytokine interleukin-6; NfL = neurofilament light; NNMF = non-negative matrix factorization; p-tau = phosphorylated tau; S100b = S100 calcium binding protein B; sTREM2 = soluble triggering receptor on myeloid cells 2; t-tau = total tau.

|  | A-T- vs A+T- | | A+T- vs A+T+ | | A-T- vs A+T+ | |
| --- | --- | --- | --- | --- | --- | --- |
|  | β_std_[95%CI] | p | β_std_ [95%CI] | p | β_std_ [95%CI] | p |
| C1 | 0.10  [-0.11,0,32] | 0.582 | **2.11**  **[1.75,2.47]** | **<0.001** | **2.21**  **[1.87,2.56]** | **<0.001** |
| C2 | 0.33  [0.08,0.58] | 0.020 | 0.48  [0.06,0.90] | 0.048 | 0.81  [0.41,1.21] | <0.001 |
| C3 | **0.49**  **[0.22,0.76]** | **<0.001** | 0.20  [-0.25,0.65] | 0.633 | 0.69  [0.26,1.12] | 0.003 |

**Supplementary Table 2 Differences on CSF components' expression by AT stages.** Standardized betas were calculated as the *β*'s divided by the standard error. Significant differences (*p*<0.05) are shown in bold.

Abbreviations: β_std_ = standardized beta; 95%CI = 95% confidence interval.


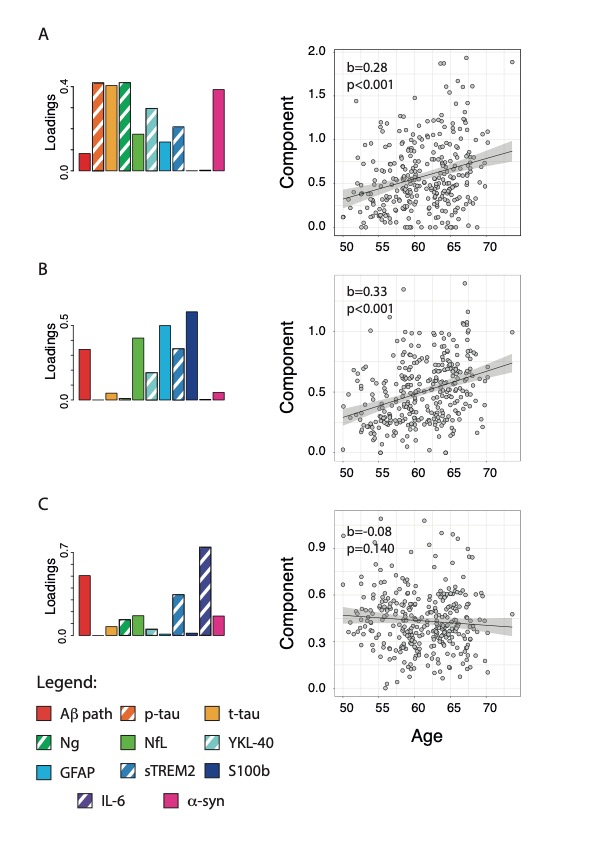


**Supplementary Figure 2 CSF components' associations with age.** The description of components' loadings is shown in the first column and expression of each component association with age in the second (first to third component: A-C). Beta estimates and p values of the association are shown in the figure. Of note, CSF Aβ42/40 levels were inverted with the aim that higher expression would represent higher Aβ pathology. Abbreviations: Aβ = amyloid-β; GM = gray matter.


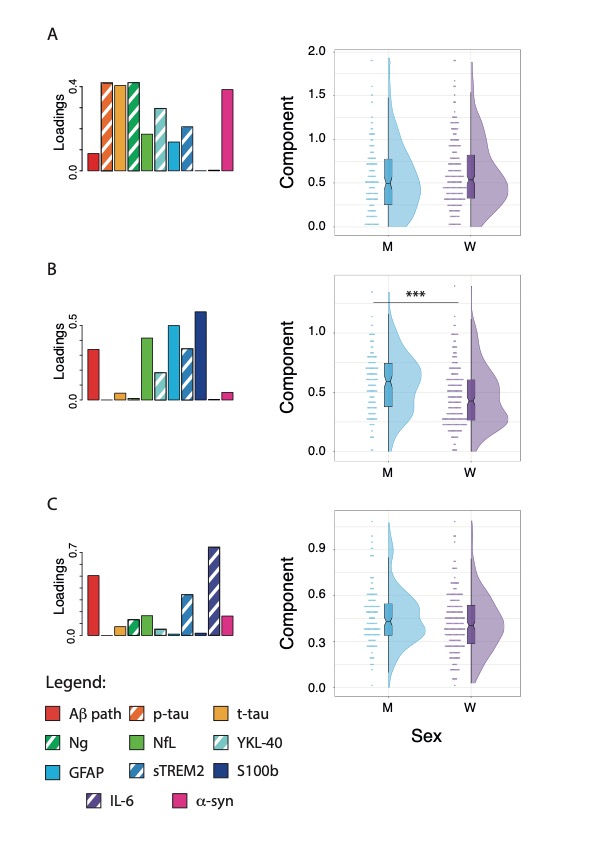


**Supplementary Figure 3 CSF components' characterization by sex.** The description of components' loadings is shown in the first column and expression of each component by sex in the second. (first to third component: A-C) Significant differences are depicted in the figure. Of note, CSF Aβ42/40 levels were inverted with the aim that higher expression would represent higher Aβ pathology. * p<0.05; ** p<0.01; ***p<0.001. Abbreviations: Aβ = amyloid-β; GM = gray matter.


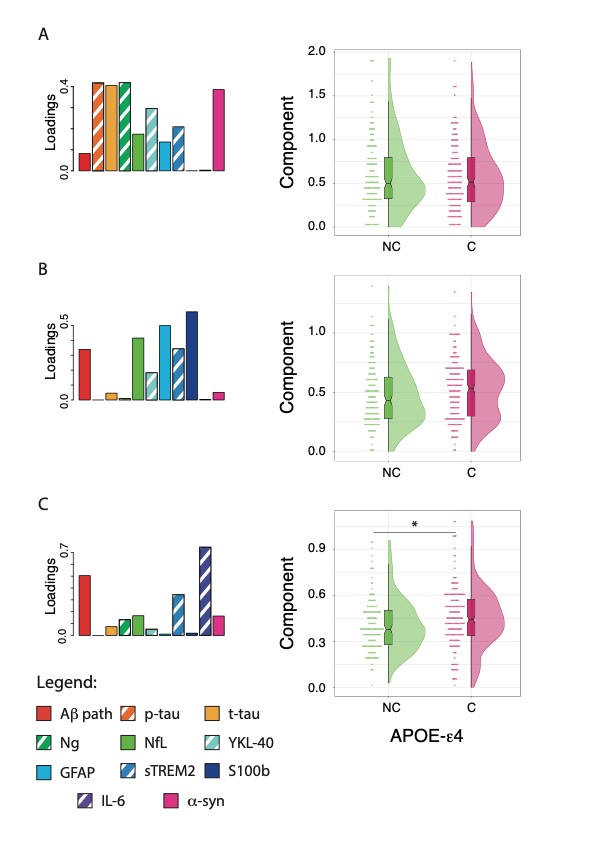


**Supplementary Figure 4 CSF components' characterization by *APOE-ε4* carriership.** The description of components' loadings is shown in the first column and expression of each component by *APOE-ε4* carriership in the second (first to third component: A-C). Significant differences are depicted in the figure. Of note, CSF Aβ42/40 levels were inverted with the aim that higher expression would represent higher Aβ pathology. * p<0.05; ** p<0.01; ***p<0.001. Abbreviations: Aβ = amyloid-β; GM = gray matter.

|  | Age | | Sex | | *APOE-ε4* carriership | |
| --- | --- | --- | --- | --- | --- | --- |
|  | β_std_[95%CI] | p | β_std_ [95%CI] | p | β_std_ [95%CI] | p |
| C1 | **0.28**  **[0.19,0.37]** | **<0.001** | 0.18  [-0.01,0.37] | 0.112 | -0.03  [-0.21,0.15] | 0.813 |
| C2 | **0.33**  **[0.25,0.42]** | **<0.001** | **-0.38**  **[-0.56,-0.21]** | **<0.001** | 0.15  [-0.02,0.33] | 0.142 |
| C3 | -0.08  [-0.17,0.01] | 0.140 | -0.19  [-0.38,-0.00] | 0.099 | **0.23**  **[0.04,0.41]** | **0.042** |

**Supplementary Table 3 Associations between CSF components' expression and age, sex and *APOE-ε4* status.** Standardized betas were calculated as the *β*'s divided by the standard error. Significant differences (*p*<0.05) are shown in bold. Men and *APOE-ε4* non-carriers were used as the reference groups.

Abbreviations: β_std_ = standardized beta; 95%CI = 95% confidence interval.

**
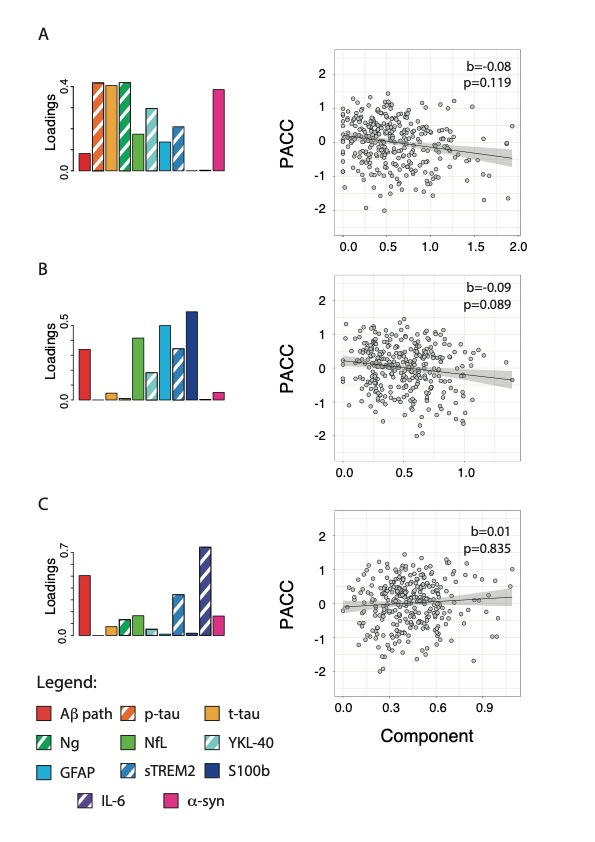
Supplementary Figure 5 CSF components' associations with cognition.** The description of components' loadings is shown in the first column and expression of each component association with PACC in the second (first to third component: A-C). Beta estimates and p values of the association are shown in the figure. Of note, CSF Aβ42/40 levels were inverted with the aim that higher expression would represent higher Aβ pathology. Abbreviations: Aβ = amyloid-β; GM = gray matter; PACC = Preclinical Alzheimer Cognitive Composite.

|  | PACC | |
| --- | --- | --- |
|  | β_std_ [95%CI] | p |
| C1 | -0.08  [-0.17,0.00] | 0.119 |
| C2 | -0.09  [-0.18,-0.00] | 0.089 |
| C3 | 0.01  [-0.07,0.09] | 0.835 |

**Supplementary Table 4 Associations between CSF components' expression and structural and metabolic AD signatures and PACC.** Standardized betas were calculated as the *β*'s divided by the standard error. Significant differences (*p*<0.05) are shown in bold.

Abbreviations: β_std_ = standardized beta; 95%CI = 95% confidence interval; PACC = Preclinical Alzheimer Cognitive Composite.

## References

1. Donohue MC, Sperling RA, Salmon DP, et al. The Preclinical Alzheimer Cognitive Composite: Measuring Amyloid-Related Decline. *JAMA Neurology*. 2014;71(8):961-970. doi:10.1001/jamaneurol.2014.803

2. Papp K v., Rentz DM, Orlovsky I, Sperling RA, Mormino EC. Optimizing the preclinical Alzheimer’s cognitive composite with semantic processing: The PACC5. *Alzheimer’s and Dementia: Translational Research and Clinical Interventions*. 2017;3(4):668-677. doi:10.1016/j.trci.2017.10.004

3. Jonaitis EM, Koscik RL, Clark LR, et al. Measuring longitudinal cognition: Individual tests versus composites. *Alzheimer’s and Dementia: Diagnosis, Assessment and Disease Monitoring*. 2019;11:74-84. doi:10.1016/j.dadm.2018.11.006

4. Höglund M, Frigyesi A. *Correspondence: Non-Negative Matrix Factorization for the Analysis of Complex Gene Expression Data: Identifi Cation of Clinically Relevant Tumor Subtypes*. www.ncbi.nlm.nih.gov/geo/,
